# Intramuscular Tranexamic Acid Administration on the Battlefield

**DOI:** 10.1155/2022/9689923

**Published:** 2022-10-13

**Authors:** David Steele, P. Kjell Ballard, Riley Burke, Brian Ferguson

**Affiliations:** ^1^Air Force Special Operations Command, USA; ^2^Emergency Medicine at Uniformed Services University of the Health Sciences, USA; ^3^University of Louisville Department of Emergency Medicine, USA

## Abstract

**Background:**

Tranexamic acid (TXA) is routinely administered intravenously (IV) and intraosseously (IO) in response to exsanguination.

**Case:**

This report describes a patient who sustained multiple high-powered rifle gunshot wounds that received battlefield-environment intramuscular (IM) administration of TXA due to inability to obtain IV / IO access. This case represents the unlikely positive outcome in the setting of multiple remarkable obstacles, which may have been ameliorated by novel administration of TXA.

**Conclusion:**

Cases of IM TXA administration as a primary intervention are not well represented in the current body of medical literature. This case report highlights a clinical scenario where IM TXA was utilized as part of first-line treatment that led to a positive clinical outcome. Although IM TXA is not yet endorsed by current trauma guidelines, this case suggests that IM route administration of TXA should be further investigated. If indeed IM administration of TXA proves just as efficacious as alternative routes, this would hold considerable advantageous implications for austere situations were sterility and IV / IO placement are impractical. This would also represent another avenue by which to decrease the time-to-TXA for patients, allowing sooner correction of hemorrhage and trauma-associated coagulopathy.

## 1. Introduction

Tranexamic acid (TXA), an antifibrinolytic agent discovered in the 1960s, is regularly used to prevent and treat bleeding complications in surgery, obstetrics, and trauma [1, 2]. In 2011, TXA was added into the Tactical Combat Casualty Care (TCCC) protocols [3]. For massive hemorrhage in trauma, TXA is delivered via intravenous (IV) or intraosseous (IO) routes. TXA is currently FDA approved for IV administrations in patients with hemophilia at risk for hemorrhage after tooth extraction and PO for the treatment of heavy menstrual bleeding. All other uses are off label. Despite this, systemic TXA has proven beneficial in postpartum hemorrhage, hemorrhage in trauma, intracranial hemorrhage, and cardiothoracic surgery [1].

Intramuscular injection (IM) is not currently FDA approved for any indication, nor is it recommended by the Committee on TCCC (CoTCCC) or the Joint Trauma Service (JTS). The current JTS Clinical Practice Guidelines (CPGs) recommend a minimum of 1-gram TXA IV, to be administered as soon as possible after the injury (with a limit of 3 hours post injury). JTS further clarifies that ideal practice is administering 1 gram within 1 hour of injury, followed immediately by a second dose of 1 gram as a drip over 8 hours [4]. These recommendations were simplified in November 2020 by the CoTCCC—now recommending 2 grams of TXA slow push. It is worth noting that all of these recommendations remain off label and are intended to guide responsible clinical judgement but have not yet been adopted as a standard practice [4].

In 2019, testing conducted on swine suggested TXA delivered via IM injection could achieve similar in vivo concentrations as those observed with IV or IO administration—even in a shock state [5, 6]. Multiple small studies have attempted to assess the viability of IM TXA in massive traumatic hemorrhage, but all have done so by giving a second dose IM after an initial IV loading dose [7, 8]. This case report describes a recent scenario where the initial TXA dose was administered IM, under fire, in a tactical environment.

## 2. Case Presentation

A midtwenties, previously healthy adult male patient presented as a casualty in care-under-fire with enemy combatants actively engaging the responding special operations medic. The patient was downed at 1132 local time after experiencing multiple gunshot wounds (GSWs) from high-powered (7.62 mm) rounds. He had taken 1 round to his chest plate, knocking him to the ground, followed by GSWs to bilateral proximal thighs, 3 GSWs to the abdomen, 1 wound to the left proximal arm, and 1 to the left chest. During the initial sweep by the paramedic, the wounds unanimously appeared consistent with entry wounds, without matched exit wounds.

The patient was obtunded upon initial evaluation with a distended abdomen and prominent evisceration. A carotid pulse was palpable, but the patient displayed agonal respirations. Effective hasty tourniquets were applied to bilateral lower extremities and the left arm. (“Hasty” application references tourniquet placement in the setting of prelimb exposure for immediate response, to ameliorate a dangerous or medically critical and time-sensitive situation that precludes full exposure of the involved limb under adequate lighting.) Next, a nasopharyngeal airway was placed. A chest seal was applied to the left chest wound; and, lastly, the evisceration was reduced into the abdominal cavity.

After rendering the treatments described above, the medic noticed that the patient had regained consciousness, and thus, he thought it is best to begin drawing up medications for pain control and hemostasis. Unfortunately, following the reduction of the evisceration, two of the three abdominal gunshot wounds displayed swift bleeding. An assaulter from the patient's team was pulled from his engagement with the ongoing gunfight to hold pressure on the abdominal wounds, while the single special forces medic drew up medication. Pressure to these wounds was initially held by a gloved hand but was rapidly replaced with combat gauze.

The team remained in a care-under-fire tactical scenario that required moving the patient 3-4 times in 8-10 minutes. Persistent fire and frequent movements eliminated time to obtain IV or IO access. The previously drawn 100 mg of ketamine and 1 gram of TXA (1000 mg/10 mL) were administered intramuscularly in the left deltoid in a conscious decision that it was likely better than no intervention at all. No immediate complications were appreciated during or after TXA administration.

Amazingly, the patient was kept alive and was handed over to medevac 10 minutes after injury (handoff at 1142), approximately 5 minutes after IM TXA administration. During the patient's 5-minute flight to the Surgical Resuscitative Team (SRT), he received an additional dose of 1-gram TXA, this time by IO (10-15 minutes post injury), with 1 unit of whole blood started. Upon arrival, the patient was rushed to the operating room (15-20 minutes postinjury). He spent 3 hours in surgery with SRT before he was stabilized for transport to a higher level of care—designated as a Role 3 capable facility. (Role 3 facilities, offering CT imaging and neurosurgical / higher specialty care capability, represent the 3^rd^ major echelon of care in the course of treatment following initial medical battlefield resuscitation (Role 1) and damage control surgery (Role 2).) During damage control surgery, the patient received 37 units of whole blood from 1253 to 1412 (local time).

The subsequent facility brought the patient back to surgery to remove abdominal packing and further control bleeding. During care, the facility exhausted blood stores in transfusing this patient, and thus, the walking blood bank was employed. In total, this patient received over 160 units of blood during the first 24-hour postinjury. Despite his substantial injuries and massive transfusion, the patient is doing well and expected to make a full recovery. At the time of this article's writing, the patient is back on limited duty and undergoing physical therapy.

## 3. Discussion

While unconventional, IM administration of TXA may have contributed to this patient's favorable outcome—and it is certainly unique as an initial measure in a care-under-fire scenario. DeSoucy et al. compared serum drug concentrations between IV, IO, and IM administration of TXA in a porcine model and found that each route of administration took 10 minutes for serum levels to reach at least 20 *μ*g/mL [5].

Regarding injection characteristics, a consensus on the effective concentration level for injections in trauma has yet to be established. Grassin-Delyle et al. used a 2X dilution (10 *μ*g/mL) from what was investigated by DeSoucy et al. in their pharmacokinetics trial. They observed that a consistent 4-11 minutes time was required to achieve therapeutic levels in human trauma patients [7]—which was consistent with that of the porcine model (using higher concentration). Regardless, drawing from this data, the administered IM TXA should have reached therapeutic concentrations in the patient at approximately the time of handover to the medical evacuation team.

The TraumaINTACT trial (for trial information: https://clinicaltrials.gov/ct2/show/NCT03875937) seeks to compare IV and IM TXA administration and pharmacokinetics in trauma patients—currently undergoing phase 2 testing. This study may provide increased clarity regarding the efficacy of administering TXA IM. Austere medicine, especially in a combat environment, may limit IV or IO access. Therefore, IM administration of TXA may drastically reduce the time to TXA administration. This would hold significant positive implications being that the value of TXA in trauma-induced coagulopathy has been well established [1, 2]. Concurrently, the time to first dose administration has been correlated with improved clinical outcomes [9].

There is logical concern that absorption might be delayed from skeletal muscle sites as a result of a shunted response in trauma (as vital organs receive priority blood flow). However, this concern cannot yet be borne out from the literature as absorption delay of IM TXA was absent in the animal model, the bleeding trauma patient, and seems immune to pharmacokinetic alterations in shock-type settings [5–7].

In addition to efficient early Tactical Combat Casualty Care (tourniquets, combat gauze, and airway adjuncts), this patient benefited from rapid transport to SRT, early administration of blood, and the availability of blood products to support a massive transfusion. All of the above played a critical role in the patient's survival and leave it unclear the extent to which the administration of IM TXA provided a meaningful intervention. However, the patient survived 15-20 minutes after injuries involving multiple large arterial bleeds. The fact that his injuries were significant enough to require 2 emergent surgeries to achieve hemorrhage control and that he received a transfusion of over 160 units of blood equally suggest that he might not have survived without the initial IM TXA dose. It can be reasonably observed that at the very least, it appears to have introduced no harm and thus might be considered where IV / IO placement are impractical.

Ideally, this case will prompt additional research for the use of IM TXA. Should IM administration prove effective in improving hemostasis, field use of self-application devices (autoinjectors) could be explored for self-aid and buddy care—further shortening time to TXA delivery and also universalizing its reach (beyond that of just medics). Though IM TXA administration is not currently CoTCCC recommended, a growing body of literature, including this case, suggests that further investigation is warranted.

## 4. Conclusion

Tranexamic acid is routinely administered IV or IO to control massive hemorrhage. IM TXA has been associated with positive results in preliminary studies and has also demonstrated rapid absorption time in patients sustaining traumatic shock. This case represents IM TXA being successfully utilized to bridge a critical patient in their initial field care to arrive alive at the surgeon's table. Although the reported case is a single instance, it highlights a positive outcome for a patient in critical condition, which merits further research. If indeed IM administration of TXA proves just as efficacious as alternative routes, this would hold considerable advantageous implications for austere situations where sterility and IV / IO placement are impractical. This would foreseeably revolutionize TXA response time, imply possible universality to TXA administration, and allow for sooner correction of hemorrhage and trauma-associated coagulopathy.
